# Case of Paraplegia

**Published:** 1868-03-15

**Authors:** Chas. M. Clark

**Affiliations:** Late Surgeon 39th Illinois vols.


### CASE OF PARAPLEGIA:

*The result of slightly depressed bone over the fronto-parietal region of skull, caused by musket-ball. Operation of trephining and cure.*

BY CHAS. M. CLARK, M.D., LATE SURGEON 39TH ILLINOIS VOLS.

M. F. S., late private of Co. E. 39th Ill. Vol. Infantry, was wounded August 15th, 1863, in front of Fort Wagner, S. C., by a conoidal ball, which struck the left side of head, impinging on the "os frontis," near the coronal suture, one inch and a half from the apex or crown of the "os frontis." Simple dressing was given at the time of the wound, and the scalp healed readily. After the scalp wound had healed, he was placed on light camp duty, and evinced no bad symptoms from continuous daily labor.

January 1st, 1864, he began to experience a feeling of numbness in his privates, nates, and right limb, which increased and extended to the left limb, and at the same time he commenced to suffer with difficulty in voiding his urine. He sought medical advice at this time, considering that he had the *gravel*, and went to a Mrs. Dr. Waiscott, Kankakee, Ill., where he was subjected to a bathing process for a period of seven (7) weeks, without any good resulting. Then he consulted Dr. Fox, of Washington, and also Dr. Johnson, who gave him an examination, and pronounced his disability to be "a general disease of the spine," resulting in paralysis. (It is due to Dr. Johnson to state that his patient, at this time, had not told him of the injury to his head.)

When treatment was commenced with Dr. F., the numbness was all confined to the right limb, but was commencing in the left; also, had lost control of his bowels, and had great difficulty in passing his water. He then tried a galvanic battery, which, as he states, "would relieve him for a few moments, and then would be as bad as before."

He went to Dr. Johnson again, and was stripped for examination. The Doctor gave him a thorough physical examination, and pronounced him sound in his "vital organs." The patient then stated to the Doctor the fact of his having been wounded in the head, and the Doctor, *after* examination, stated that an *operation* would be necessary in order to relieve him of his trouble.

At this time, a Dr. Rainford, of Kankakee county, wished him to remain and be experimented upon with the galvanic battery and air-pump, holding forth inducements of permanent benefit.

The case was brought to me November 27th, 1867, for examination, and after a thorough inspection, it was decided to operate by raising the portion of depressed skull as a means of restoration to a normal condition.

December 9th, 1867, he was placed under the influence of a mixture of chloroform and ether — the hair shaved from the part, and a conical incision made through the scalp, and the flaps turned back. The periosteum was then scraped off and the trephine applied at a point that would cover the whole of the depressed bone. He was allowed to recover from the anæsthetic before the section was complete, in order that when the bone was raised we might note more fully the result.

As soon as the circle of bone was raised, (which was one inch in diameter), he commenced the free use of a limb that had hitherto been useless, and expressed himself as feeling as "good as ever!" Sensation and motion had returned almost on the instant that the bone was lifted, and he kicked about joyously.

After dressing the wound he walked unaided to his bed.

December 10th. Is feeling very comfortable this morning. Advised a mild aperient, with a pulvis of *Opii et Potassæ Nitrat.* Cold water dressing to the head.

11th. Rested well last night, but this morning had a light chill. Gave *Quinine* and *Dover's powder*.

12th. Expresses himself as feeling first-rate. Sensation and motion have returned almost complete, although there is some numbness about the perineum, but has no difficulty in controlling his urine or fæces. Removed stitches from the wound, which has united, excepting at the points of incision, where there is slight suppuration.

13th. Sleeps well at night; bowels move off naturally, and can control his water, which passes freely. Had some little vertigo in the morning, but soon passed away.

14th to 23rd. Is increasing in strength daily, and can walk easily and readily without help of cane—especially up and down stairs. Wound of scalp entirely healed.

24th. Went home to spend the holidays, and walked nearly three miles.

January 3rd, 1868. Returned to my care; has taken some cold, and feels weak; placed him on *Iron* and *Quinine*.

4th. The medicine gives him vertigo, and he loses control of his bowels and bladder.

5th to 10th. Discontinued the *Iron* and *Quinine*, and gave him *Brom. potass.* and the best of food.

13th. Came to my office to-day, saying that he feels perfectly well, and wants a plate fitted over the "hole in his head." Says that his animal passions have returned, and he wishes to go home to his wife. Has had no passion for a woman before since January, 1867, at which time the parts became paralyzed.

14. Went home.

It should have been stated, that the least pressure over the depressed portion of bone gave him very peculiar sensations down to the extremities of the toes of right foot.

When the skull was fully exposed and denuded of periosteum, the bone immediately over the depression seemed loose in texture, and blood oozed freely from it. The circle of bone raised was $\frac{2}{8}$ of an inch in thickness throughout its whole area.

